# Blood protein profiles related to preterm birth and retinopathy of prematurity

**DOI:** 10.1038/s41390-021-01528-0

**Published:** 2021-04-24

**Authors:** Hanna Danielsson, Abdellah Tebani, Wen Zhong, Linn Fagerberg, Nele Brusselaers, Anna-Lena Hård, Mathias Uhlén, Ann Hellström

**Affiliations:** 1 Department of Microbiology, Tumor and Cell Biology, Centre for Translational Microbiome Research, Karolinska Institutet, Stockholm, Sweden; 2 Sach’s Children’s and Youth Hospital, Södersjukhuset, Stockholm, Sweden; 3 Science for Life Laboratory, Department of Protein Science, KTH—Royal Institute of Technology, Stockholm, Sweden; 4 Department of Metabolic Biochemistry, Rouen University Hospital, Rouen, France; 5 Normandie Univ, UNIROUEN, CHU Rouen, INSERM U1245, Rouen, France; 6 Global Health Institute, Antwerp University, Antwerp, Belgium; 7 Department of Head and Skin, Ghent University, Ghent, Belgium; 8 The Institute of Neuroscience and Physiology, Sahlgrenska Academy, University of Gothenburg, Sahlgrenska University Hospital, Gothenburg, Sweden

## Abstract

**Background:**

Nearly one in ten children is born preterm. The degree of immaturity is a determinant of the infant’s health. Extremely preterm infants have higher morbidity and mortality than term infants. One disease affecting extremely preterm infants is retinopathy of prematurity (ROP), a multifactorial neurovascular disease that can lead to retinal detachment and blindness. The advances in omics technology have opened up possibilities to study protein expressions thoroughly with clinical accuracy, here used to increase the understanding of protein expression in relation to immaturity and ROP.

**Methods:**

Longitudinal serum protein profiles the first months after birth in 14 extremely preterm infants were integrated with perinatal and ROP data. In total, 448 unique protein targets were analyzed using Proximity Extension Assays.

**Results:**

We found 20 serum proteins associated with gestational age and/or ROP functioning within mainly angiogenesis, hematopoiesis, bone regulation, immune function, and lipid metabolism. Infants with severe ROP had persistent lower levels of several identified proteins during the first postnatal months.

**Conclusions:**

The study contributes to the understanding of the relationship between longitudinal serum protein levels and immaturity and abnormal retinal neurovascular development. This is essential for understanding pathophysiological mechanisms and to optimize diagnosis, treatment and prevention for ROP.

**Impact:**

- Longitudinal protein profiles of 14 extremely preterm infants were analyzed using a novel multiplex protein analysis platform combined with perinatal data.
- Proteins associated with gestational age at birth and the neurovascular disease ROP were identified.
- Among infants with ROP, longitudinal levels of the identified proteins remained largely unchanged during the first postnatal months.
- The main functions of the proteins identified were angiogenesis, hematopoiesis, immune function, bone regulation, lipid metabolism, and central nervous system development.
- The study contributes to the understanding of longitudinal serum protein patterns related to gestational age and their association with abnormal retinal neuro-vascular development.

## Introduction

Around the world, nearly one in ten children is born preterm, i.e., born before 37 completed weeks of gestation.^1^ Being born too early is often dangerous or even fatal. Preterm birth and the following complications are still the second most common cause of death in children under the age of 5 years.^2^ The survivors are facing many challenges, especially during the first months of life. The degree of immaturity is often a determinant of the infant’s health. Extremely preterm infants, born at a gestational age (GA) of <28 weeks, have significantly higher morbidity and mortality than term infants.^3^ One of the diseases mainly affecting extremely preterm infants due to immaturity is retinopathy of prematurity (ROP). ROP is a multifactorial neurovascular disease affecting the immature retina and its vasculature, and may lead to retinal detachment and blindness.^4^ Conflicting numbers have been reported about the prevalence of ROP in extremely preterm infants. The occurrence of any stage of ROP ranges from 5 to 73%. This notable range might reflect both neonatal care and survival in different regions as well as varying diagnostic routines.^4–10^

The causes of ROP are still not completely understood. The first cases were seen in the 1940s following excessive oxygen treatment of preterm infants, and since then researchers and neonatologist have strived to find the optimal balance of oxygen supply.^11,12^ Too little oxygen might be harmful and decrease the chance of survival; too much oxygen may increase the risk of ROP and blindness.^13–16^ In addition, low GA and birth weight, decreased postpartum levels of insulin growth factor-1, impaired blood glucose control, insufficient nutrition such as lack of ω-3 and ω-6 long-chain polyunsaturated fatty acid, and infections have been associated with the development of ROP.^4^

Recently, the potential of exploratory omics to increase the knowledge of preterm birth and ROP has been highlighted.^17–21^ These studies display the power of extensive protein profiling as a tool for future precision medicine. Nonetheless, follow-up studies are required to confirm the targets for either therapeutic or diagnostic purposes. The possibility to get detailed information from a single drop of blood about hundreds of proteins at the same time can be used to discover previously unknown connections between physiology and diseases such as ROP. If the first months of life could be better understood, and in particular the mechanisms of immaturity, this would lead to improvements in the neonatal intensive care to facilitate a more normal development and a healthier start of life for preterm infants. In this study, we have performed longitudinal multiplex protein analysis in 14 extremely preterm infants born at GAs 22–27 weeks to investigate the associations of blood protein levels with GA and ROP.

## Methods

### Patients and nutritional management

The current study was based on longitudinal blood samples from the Donna Mega Study, which was a randomized, open-label, controlled trial conducted at a single site in Sweden comparing the effect of two parenteral fatty acid solutions.^22^ The protocol for the Donna Mega study is available at clinicaltrial.gov (Clinical trial NCT 02760472). The Regional Ethical Board in Gothenburg (Dnr 303-11) approved the study. Informed written consent was obtained for all participants from their parents or guardians. The major outcome of the Donna Mega Study was to investigate the impact of parenteral nutrition with and without fish oil on preterm morbidities. The nutritional strategy has been described previously.^22,23^ Infants born at GA of <28 weeks (based on ultrasonography examination dating) in the neonatal intensive care unit at Sahlgrenska University Hospital in Gothenburg, Sweden, from April 2013 to September 2015 were investigated. Seventy-eight out of 90 individuals fulfilled the criteria for final evaluation, surviving to 40 weeks postmenstrual age (PMA).

### Study cohort and blood sampling

**Fig. 1:**
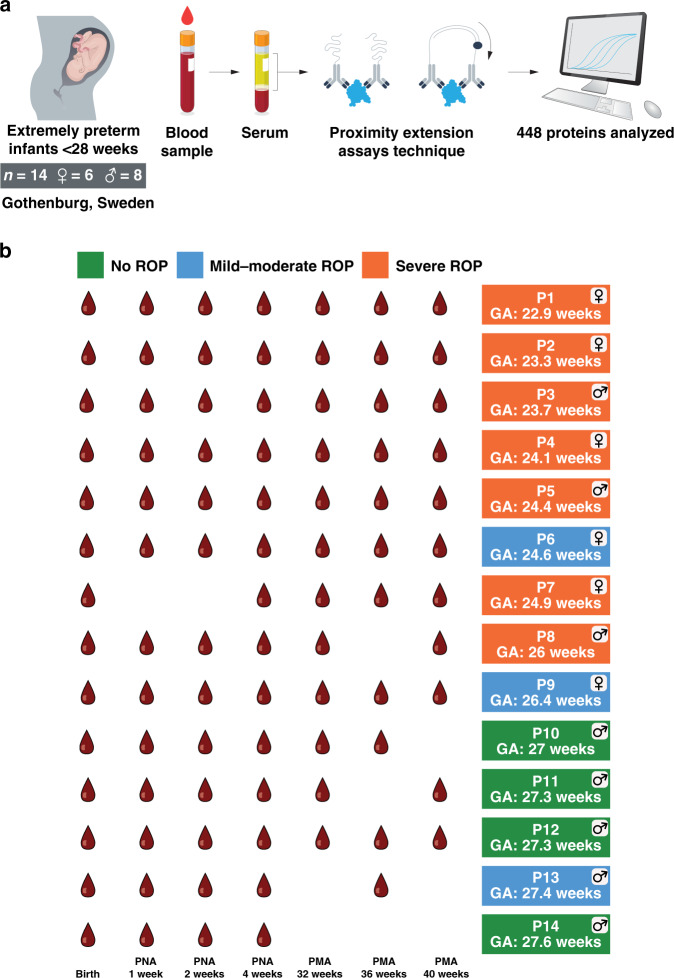
Schematic description of the study and study cohort. **a** The cohort consisted of 14 extremely preterm infants (six females) born before 28 weeks of gestation in Gothenburg, Sweden. Blood samples were centrifuged to serum and levels of 448 unique protein targets in each sample were determined using the proximity extension assay technique. Clinical variables were integrated with the serum protein profiles. **b** Blood samples were collected at birth, after 1, 2, and 4 weeks postnatal age (PNA), and weeks 32, 36, and 40 postmenstrual age (PMA)). In total, 88 samples from 14 individuals were analyzed. The gestational ages varied from 22.9 to 27.6 weeks. Seven infants had severe retinopathy of prematurity (ROP) defined as stage 3 or type 1 ROP. Three had mild-to-moderate ROP defined as stages 1 and 2 and four had no signs of ROP.

For the current study, the cohort consisted of 14 selected extremely preterm infants (six girls) with a distribution of GA from 22.9 to 27.6 gestational weeks (Fig. 1a). Birth weights varied from 415 to 1235 g. Four children were not affected by ROP, three had mild-to-moderate ROP, and seven had severe ROP, of whom six were treated with laser (Fig. 1b). GA, weight, sex, delivery mode, and other diseases are shown in Supplementary Table S1 (online). Coordinated with clinical relevance, up to seven blood samples were collected at postnatal days 1, 7, 14, and 28 and at PMA 32, 36, and 40 weeks (see Fig. 1a, b). The blood samples were analyzed using proximity extension assays and clinical variables were linked to the serum protein profiles.

### Eye examinations for ROP diagnostics

ROP screening started at 6 weeks postnatal age, but not before 31 weeks PMA.^24^ Retinal examinations were thereafter performed every week to every second week, or with individual intervals, depending on ROP severity, until the retina was fully vascularized or the condition was considered stable. The vast majority of the retinal examinations were performed by one ophthalmologist (author A.H.) at the same neonatal intensive care unit at Sahlgrenska University Hospital in Gothenburg, Sweden. ROP was categorized according to the international classification as no ROP, mild/moderate ROP (stages 1 and 2), and severe ROP (stage 3/type 1 ROP).^25^

### Serum protein profiling

Multiplex extension proximity assay technology was used for serum protein analysis (Olink Bioscience, Uppsala, Sweden).^20,26^ As previously reported in our recent study by Zhong et al.,^20^ using microliter plates where each well contained 96 pairs of DNA-labeled antibody probes, 92 protein biomarkers could be measured in 88 samples. The data were normalized using both an internal control (extension control) and an inter-plate control and then transformed using a predetermined correction factor to minimize intra- and inter-run variation. The preprocessed data were provided in the arbitrary unit Normalized Protein Expression (NPX) on a log 2 scale and where a high NPX represents a high protein level. For the current study, a total number of 460 protein assays were analyzed using five Olink panels, including Cardiometabolic, Cardiovascular II, Cardiovascular III, Development, and Metabolism. After removal of the samples that did not pass the quality control and normalization, a total of 448 unique proteins from 88 blood samples were analyzed, see Supplementary Table S2 (online).

### Hierarchical clustering and PCA analysis

For clustering, the expression profiling of each protein was first standardized with a standard deviation of 1 centered at 0. The scaled values from all 88 samples were used to create the Euclidean distance matrix for dendrogram generation. Dendrograms showing gene expression in heatmaps have been clustered using the Ward2 algorithm, an implementation of Ward’s minimum variance method implemented as “Ward.D2” in R package pheatmap.^27,28^ Principal component analysis (PCA) has been performed using the R package pcaMethods with default parameters.^29^ Spearman’s correlation (Spearman’s *ρ*) was used to compute the pairwise correlation.^30^ Multiple testing correction has been performed using the Benjamini–Hochberg method.^31^

## Results

### Description of the study

A schematic overview of the study and ROP outcomes are shown in Fig. 1a, b. All infants were sampled for blood serum up to seven time points from birth to term-equivalent age, e.g., 40 weeks PMA (see above).

### Results of the protein profiling

**Fig. 2:**
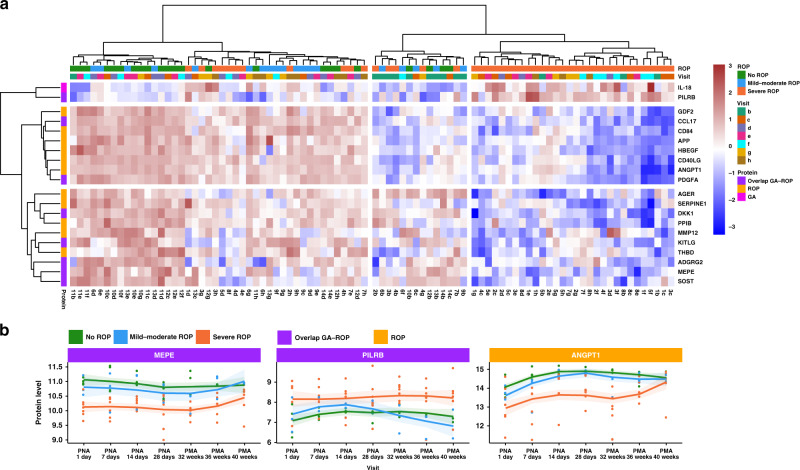
Overview of the results including examples of longitudinal protein levels. **a** A heatmap demonstrating levels of the 20 proteins most significantly correlating to gestational age (GA) and/or retinopathy of prematurity (ROP). Visits and clustering from ROP stage are also shown. **b** Longitudinal results for the proteins matrix extracellular phosphoglycoprotein (MEPE), paired immunoglobulin-like type 2 receptor beta (PILRB), and angiopoietin-1 (ANGPT1) show distinctions in protein levels between the infants with different stages of ROP. MEPE and PILRB are both correlated to both GA and ROP and ANGPT1 correlated to ROP. The longitudinal results of all the 20 most correlating proteins are shown in Supplementary Figure S1 (online).

The blood samples (*n* = 88) were analyzed using proximity extension assays using five panels of altogether 448 unique target proteins. The panels included proteins involved in inflammation, neurogenesis, cardiovascular disease, and cellular metabolism. The assay has the advantage that only minute sample volumes (<5 μl) are needed, which is a great advantage when analyzing extreme preterm infants. Results of all targets in the 88 samples are listed in Supplementary Table S2 (online). In Fig. 2a, an overview of the results has been visualized as a heatmap showing the most significant proteins (*n* = 20, cut-off >0.5) correlating with GA and/or ROP across all the analyzed samples. A clear pattern can be observed with most of the samples from the children with ROP clustering together. Many of these proteins showed lower blood levels for several months as compared to the non-ROP infants. Only one protein, paired immunoglobulin-like type 2 receptor beta (PILRB), showed the opposite trend with higher levels in the ROP group (Fig. 2b and Supplementary Figure S1 (online)).

Most antecedent studies have focused on one or two timepoints for proteomic measurements in relation to preterm birth. However, we believe that development during the first months in life can be better understood if the gradual maturation can be described. Therefore, we present longitudinal proteomic measurements. In infants with lower GA at birth and more severe ROP, meaning more severe abnormal neurovascular development, the protein levels started at lower levels and remained low during the first months in life for a majority of the correlating proteins (*n* = 18/20). However, ~32–40 weeks PMA increasing expressions were observed in several proteins towards similar levels as the more mature infants with normal neurovascular development. This is illustrated by proteins matrix extracellular phosphoglycoprotein (MEPE) and angiopoietin-1 (ANGPT1) in Fig. 2b and in Supplementary Figures S1, S2, and S4 (online).

### Proteins correlating with GA and ROP

**Fig. 3:**
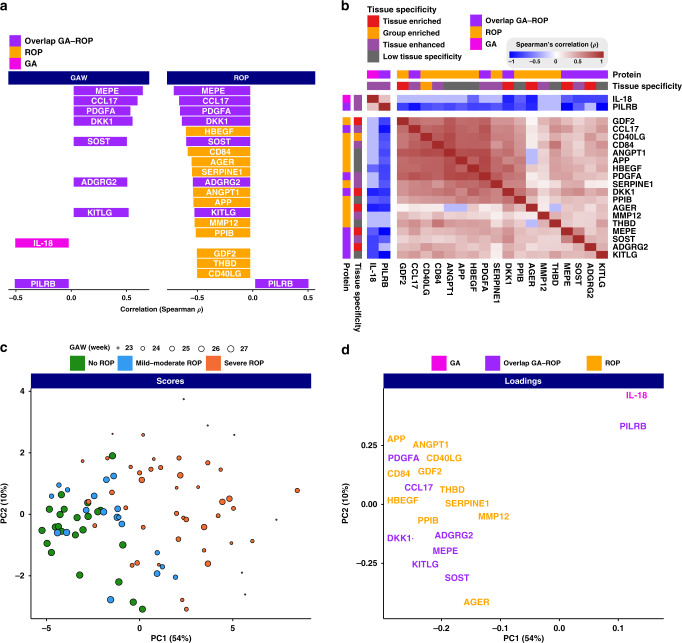
Correlation to gestational age (GA) and retinopathy of prematurity (ROP), co-expression, and protein profiles depending on ROP status for the 20 most significantly correlated proteins. **a** Positive or negative correlation to GA and/or ROP. Most proteins positively correlated to GA is negatively correlated to ROP; the size of the box corresponds to the level of correlation on the *x*-axis. **b** Co-expression analysis showing the correlation in-between the individual proteins. Two main clusters were observed. **c** Principal component analysis (PCA) demonstrated clustering trends regarding GA and stage of ROP. Each individual blood sample has a distinct protein profile depending on the infants’ GA at birth and later ROP development. **d** The loadings plot of the PCA shows the relationships between covarying proteins and demonstrates the certain proteins underlying the sample-related clustering patterns.

To extend our understanding of the proteome changes in preterm birth and the development of ROP, we assessed the correlation of blood proteins with GA and ROP. Of the 448 analyzed proteins, 20 showed significant correlations with GA, ROP, or both as illustrated in Fig. 3a. Among them, interleukin-18 (IL-18) and PILRB were negatively correlated with GA, whereas seven proteins had a positive correlation, including adhesion G-protein-coupled receptor G2 (ADGRG2), C–C motif chemokine 17 (CCL17), dickkopf-related protein 1 (DKK1), kit ligand (KITLG), MEPE, platelet-derived growth factor subunit A (PDGFA), and sclerostin. The majority of the proteins above (8/9) were also correlated to ROP in addition to GA (Fig. 3a). Among them, PILRB was the only protein found to be positively associated with severe ROP. The remaining seven proteins were negatively associated with severe ROP. Moreover, 11 additional proteins with varying functions were associated with only ROP as presented in Fig. 3a.

To explore the co-expression between these 20 proteins, Spearman’s correlation analysis was performed and the results are shown in Fig. 3b. The results yielded two main clusters, one includes only IL-18 and PILRB, and the other includes all the remaining proteins. According to the Human Protein Atlas database (www.proteinatlas.org), five of the 20 proteins are known to have a tissue-enriched expression with higher levels in a single tissue as compared to all other analyzed tissues, and these are ADGRG2 (epididymis), advanced glycosylation end product-specific receptor (AGER) (lung), DKK1 (placenta), growth/differentiation factor 2 (GDF2) (liver), and MEPE (brain). To get a holistic unsupervised overview of the samples, we performed a PCA based on the serum profiles of all proteins. The scores plotted in Fig. 3c show the clustering trends regarding the three ROP groups along with the first principal component (PC1). Indeed, similar samples are close to each other based on their protein profile similarity. This PCA analysis suggests that each individual blood sample has a distinct protein profile depending on the infants’ GA at birth and later ROP development. The infants born at later GAs with less ROP cluster to the left, while the more immature infants with lower GA and severe ROP cluster to the right. Furthermore, the loadings plot shown in Fig. 3d highlights the driving proteins that underlie the sample-related clustering patterns supporting the patterns observed in Fig. 3b. In summary, the blood proteome profiling analysis described here shows that blood protein levels correlating with ROP, GA, or both could be identified.

### Functions of the GA- and ROP-associated blood proteins

**Fig. 4:**
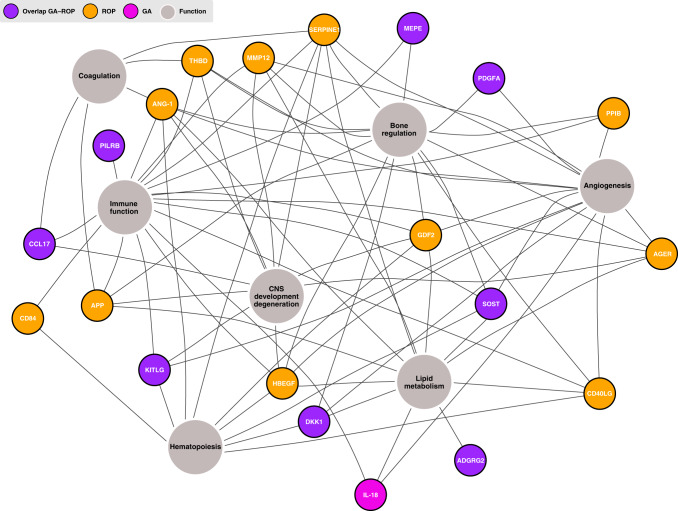
Network graph illustrating the areas of function in gestational age (GA) and/or retinopathy of prematurity (ROP)-associated serum proteins. The proteins are described to be functioning within seven main areas, such as angiogenesis hematopoiesis, immune function, and bone regulation.

To increase the knowledge of the physiological involvement of the proteins correlating with GA and/or ROP, their functions were investigated. The literature was scrutinized for protein functions in PubMed using search terms combining the protein denominations or abbreviations and functionality terms. In Fig. 4, a network-based summary of the main functions of each protein is presented. The proteins identified to be correlated to ROP showed connections to functions central for the development of retinopathy, such as angiogenesis, neurogenesis, osteogenesis, immune functions, and also lipid metabolism. All 11 proteins are involved in immune function: AGER, ANGPT1, amyloid-beta precursor protein (APP), CD40 ligand (CD40LG), SLAM family member 5 (CD84), GDF2, heparin-binding EGF-like growth factor (HBEGF), macrophage metalloelastase (MMP12), peptidyl-prolyl *cis*–*trans* isomerase B (PPIB), plasminogen activator inhibitor 1 (SERPINE1), and thrombomodulin (THBD).^32–45^ The majority (9/11) operate in physiologic and pathologic angiogenesis: AGER, ANGPT1, CD40LG, GDF2, HBEGF, MMP12, PPIB, SERPINE1, and THBD.^46–57^ ANGPT1, CD40LG, CD84, GDF2, HBEGF, and SERPINE1 acts in hematopoiesis.^58–62^ AGER, ANGPT1, APP, GDF2, HBEGF, MMP12, SERPINE1, THBD, and possibly also PPIB are shown to be involved in the central nervous system (CNS) development or CNS degeneration.^32,39,52,63–67^ Further on, ANGPT1, APP, SERPINE1, and THBD acts in coagulation,^45,48,68,69^ AGER, ANGPT1, APP, CD40LG, GDF2, HBEGF, MMP12, and SERPINE1 in lipid metabolism^52,70–76^ and AGER, ANGPT1, APP, CD40LG, GDF2, HBEGF, MMP12, PPIB, SERPINE1, and THBD in bone regulation.^32,52,77–89^ The proteins correlating to both GA and ROP show similar functions.^90–110^ IL-18, the protein correlating to only GA and not ROP, is mainly active in immune function but is also described to be involved in angiogenesis and fat metabolism.^111–113^ These results confirm the involvement of impaired angiogenesis, neurogenesis, and immune function in ROP development, but also demonstrated the association with perhaps less expected proteins involved in lipid and bone metabolism.

## Discussion

In this study, we present proteins associated with GA, ROP, or both, with most of these proteins not previously described to be related to clinical immaturity and impaired neurovascular development. Furthermore, in infants with severe ROP, we found consistently reduced protein levels in a majority of the correlating proteins in the first months of life, suggesting an inability of physiological regulation associated with disease development. These longitudinal patterns give us important clues to understanding the extrauterine development and maturation of preterm infants. It is well known that GA is a major risk factor for ROP, but the remaining disease mechanisms are still not completely understood. We here demonstrate eight proteins with overlapping correlation, but also 11 proteins correlating to only ROP but not GA, implying a true protein–disease connection more than merely an indication of GA (Figs. 2–4). This suggests that these proteins might be used as potential biomarkers for predicting ROP, although this needs to be validated in a larger cohort.

Many of the proteins are involved in angiogenesis, immune function, lipid metabolism, coagulation, neurogenesis, and osteogenesis, which further sheds light on the physiological mechanisms in the development of ROP.

The current knowledge of protein functions is mainly derived from experimental studies on animals or cultured cells. Some of these proteins are secreted and others are attached to cell surfaces or intracellular membranes, while some have both a cell-bound and a soluble form, sometimes with different biological roles. As mentioned above, our findings demonstrate that infants with severe ROP have sustained reduced protein levels in the first months of life, with a slow rise starting around week 32 PMA. We have earlier described how dramatic changes in protein levels were seen at the first week of life in the same infants with many of the proteins changing already during the first week had a liver origin.^20^ Here, we believe that the initial persisting low protein levels in infants severely affected by ROP suggest an inability to activate the functions needed for normal development, which leads to dysfunctional neurovascular development. For some proteins, the low expressions were followed by an increase, which started around PMA 32 weeks among the subjects most severely affected by ROP. In the development of severe ROP, the first phase of interrupted vascularization starts from birth and is normally visualized by retinal examinations from around week 32 PMA, when the second phase of ROP starts.^4^ The increased protein expression might be due to the active pathological angiogenesis in ROP phase 2. We present this pattern for ANGPT1, APP, CD84, CCL17, KITLG, MEPE, PDGFA, DKK1, CD40LG, and SERPINE1 proteins, of which the functions are mostly associated with angiogenesis, immune function, neurogenesis, hematopoiesis, and bone and lipid metabolism. This model of initial decreased expression followed by increased levels for proteins important for retinal development is in line with theories also discussed by Lynch et al.,^18^ although they included only one sample for analysis, from the first week in the life. The specific proteins identified as most interesting by Lynch et al., such as superoxide dismutase (Mn), mitochondrial (MnSOD), proprotein convertase subtilisin/kexin type 9, and insulin-like growth factor-binding protein-7, have been examined, but not found significant in our analysis. Moreover, Markasz et al.^21^ have recently suggested the association of eight proteins, measured at postnatal age 2 days, with the later development of ROP. We have analyzed five of these proteins and cannot confirm a correlation to ROP or GA at birth. Altogether, our findings imply an inability of regulation, leading to insufficient protein production initially followed by an increase at a time point where unphysiologic neovascularization occurs, i.e., severe ROP. Whether these abnormal levels are causing the disease, are a consequence of it, or only display an association remains to be decided.

Many of the proteins found here that correlate negatively to ROP are as of today not used for diagnostics of ROP. Nonetheless, some of the proteins have been acknowledged for the possible involvement in diabetic retinopathy or ROP: AGER, ANGPT1, CD40LG, GDF2, MMP12, SERPINE1, THBD, DKK1, PDGFA, and IL-18.^47,50,51,54,56,58,92,101,114–123^ For example, Lamine et al.^115^ presented recently that CD40LG is associated to both occurrence and severity of type 2 diabetic retinopathy, which shares some physiological connections with ROP. Interestingly, many of the identified proteins are related to both development and degeneration, representing impaired function at both ends of the lifespan. This is illustrated by the association of APP to both neurogenesis and neurodegenerative diseases as well as the correlation of ANGPT1 with both ROP and diabetic retinopathy^58,64,114,116,124–126^. Further on, there is a clear association between osteogenesis and bone metabolism, a mechanism one can believe is connected to hematopoiesis. The connection to lipid metabolism, which is mainly among the proteins correlated to ROP, is likely due to the close connection between angiogenesis and lipid metabolism. Recently, the association between ROP and anemia as well as with thrombocytopenia has been stressed^127,128^. Our results strengthen the conclusion that angiogenesis and neurogenesis, and also osteogenesis and its link to hematopoiesis and coagulation is of utmost importance for understanding and preventing ROP in preterm infants.

It is important to point out that the study included a limited number of preterm infants who were individually selected in order to represent minimum and maximum stages of ROP as well as a wide range of GA <28 weeks at birth. The reason this selection was performed was to, in this pilot study, optimize the identification of possible differences in protein profiles in relation to ROP development and degree of immaturity. Nonetheless, it is the first to relate high-resolution temporal protein profiles in association with GA and ROP. Most previous research in this field has performed analysis restricted to one or limited timepoints. We present a unique, longitudinal sampling resulting in a detailed insight into the protein changes over time. The longitudinal approach revealed correlations not discoverable by intermittent sampling. Further on, previous preterm proteomic analyses have often used cord blood. However, recently, the correspondence in proteomics of peripheral blood and cord blood has been disputed.^17^ As the technical comprehensive and sensitive protein profiling platform used here only requires a few microliters of blood, a follow-up study involving a substantially larger cohort to validate the protein profiles is ethically doable and is on its way in the clinical trial Mega Donna Mega (ClinicalTrials.gov Identifier: NCT03201588). This follow-up will also include a comparison of proteomics for preterm cord blood and peripheral blood to shed light on the differences in proteomic expression. Although interpretations must be cautious as yet due to the limited number of selected individuals, we have identified potential biomarkers to predict which children develop ROP. Such a prediction model would be a major advance in the diagnostics and treatment of preterm infants.

In conclusion, proteins associated with both the degree of immaturity and ROP were identified. These proteins have never before in clinical studies been linked to preterm birth or ROP and their connection is highly interesting since their functions such as angiogenesis, CNS development, immune function, osteogenesis, lipid metabolism, coagulation, and hematopoiesis are partly novel to the field. This opens possibilities to further study the relationship between these areas, immaturity, and ROP. Interestingly, when adjusting for GA at birth, we identified some proteins showing correlation with ROP and not with GA at birth, suggesting that these proteins have a role in pathologic angiogenetic development beyond mere immaturity. A majority of these proteins were low at birth and remained low during the first postnatal months followed by an increase in near-term-equivalent age, demonstrating an initial inability of these infants to increase their production. This illustrates that for certain proteins there might be a postnatal destiny pattern depending on the degree of immaturity, resulting in immaturity-linked diseases. We also see possibilities for a predictive model for ROP. Further knowledge and potential interventions regarding these proteins might be a possibility for future prevention of abnormal neurovascular development.

## Supplementary information

Supplementary Materials

Supplementary Tables

## Data Availability

All the data used in the study are available in the Supplementary Material.
