# Supplementary material for: Blood protein profiles related to preterm birth and retinopathy of prematurity
Source: Pediatr Res. 2021 Apr 24;91(4):937–46. doi: 10.1038/s41390-021-01528-0 (PMC9064798; doi:10.1038/s41390-021-01528-0)
Supplement: Supplementary file 1 — Supplementary Materials [file 41390_2021_1528_MOESM1_ESM.docx]

**Supplementary Materials**

Blood protein profiles related to preterm birth

and retinopathy of prematurity

Hanna Danielsson^1,2^**^#^**, Abdellah Tebani^3,4,5^**^#^**, Wen Zhong^3^, Linn Fagerberg^3^, Nele Brusselaers^1,6,7^, Anna-Lena Hård^8^, Mathias Uhlén^3^ and Ann Hellström^8*^

^1^ Centre for Translational Microbiome Research, Department of Microbiology, Tumor and Cell Biology, Karolinska Institutet, Stockholm, Sweden.

^2^ Sach's Children's and Youth Hospital, Södersjukhuset, Stockholm, Sweden.

^3^ Science for Life Laboratory, Department of Protein Science, KTH - Royal Institute of Technology, Stockholm, Sweden.

^4^ Department of Metabolic Biochemistry, Rouen University Hospital, 76000 Rouen, France.

^5^ Normandie Univ, UNIROUEN, CHU Rouen, INSERM U1245, 76000 Rouen, France.

^6^ Global Health Institute, Antwerp University, Belgium.

^7^ Department of Head and Skin, Ghent University, Belgium.

^8^ The Institute of Neuroscience and Physiology, Sahlgrenska Academy, University of Gothenburg, Sahlgrenska University Hospital, Gothenburg, Sweden.

**^#^**Contributed equally

*To whom correspondence should be addressed: Prof Ann Hellström, Department of Clinical Neuroscience, Institute of Neuroscience and Physiology, Sahlgrenska Academy, University of Gothenburg, S-416 85 Gothenburg, Sweden. Phone: +46 8 768979196. Email: ann.hellstrom@medfak.gu.se

**Supplementary Figure S1 (online). Longitudinal blood protein levels.** Results of the 20 most significantly correlating proteins, highlighted according to ROP-status.

**Supplementary Figure S2 (online).** **Longitudinal blood protein levels.** Results of the gestational age-related proteins highlighted according to three prematurity stages; lower than 24 weeks (orange), between 25-26 weeks (blue) and higher than 27 weeks (green)**.**

**Supplementary Figure S3 (online).** **Correlation scatter plot.** Correlation between ROP-related proteins and ROP stages.

**Supplementary Figure S4 (online).** **Longitudinal blood protein levels.** Results of the 20 most significantly correlating proteins, highlighted according to ROP-status along postnatal age.

**Supplementary Figure S5 (online).** **Principal Component Analysis (PCA).** a) PC1 loadings plot of the 20 most significantly correlating proteins. b) PC1 scores plot of the analyzed samples colored according to their ROP-status.

**Supplementary Table S1 (online).** **Characteristics and morbidity of the subjects.**

**Supplementary Table S2 (online). Serum proteome profiling of fourteen extremely preterm babies**.

**Supplementary Table S3 (online).** **Spearman correlation analysis of gestational age and retinopathy of prematurity with the assessed 448 serum proteins.**

**Supplementary Table S4 (online).** **Spearman correlation analysis between the 448 serum proteins.**

**Supplementary Figure S1 (online). Longitudinal blood protein levels.** Results of the 20 most significantly correlating proteins, highlighted according to ROP-status.

**Supplementary Figure S2 (online).** **Longitudinal blood protein levels.** Results of the gestational age-related proteins highlighted according to three prematurity stages; lower than 24 weeks (orange), between 25-26 weeks (blue) and higher than 27 weeks (green)**.**

**Supplementary Figure S3 (online).** **Correlation scatter plot.** Correlation between ROP-related proteins and ROP stages.

**Supplementary Figure S4 (online).** **Longitudinal blood protein levels.** Results of the 20 most significantly correlating proteins, highlighted according to ROP-status along postnatal age.

**Supplementary Figure S5 (online).** **Principal Component Analysis (PCA).** a) PC1 loadings plot of the 20 most significantly correlating proteins. b) PC1 scores plot of the analyzed samples colored according to their ROP-status.

**Supplementary Table S1 (online).** **Characteristics and morbidity of the subjects.**

| **ID** | **GA***  **(weeks)** | **BW ***  **Gram**  **(SDS*)** | **Sex** | **Delivery mode** | **Morbidity**** |
| --- | --- | --- | --- | --- | --- |
| 1 | 22,86 | 530  (-0.46) | Male | Vaginal | BPD, Cholestasis, NEC, PDA, Sepsis, ROP (stage 3) |
| 2 | 23,29 | 585  (-0,32) | Male | Vaginal | IVH (grade 2), PDA,  ROP (stage 3) |
| 3 | 23,71 | 460  (-2,05) | Female | Vaginal | BPD, PDA, Sepsis,  ROP (stage 3), |
| 4 | 24,14 | 650  (-0,52) | Male | Vaginal | BPD, IVH (grade 4), PDA, Sepsis, ROP (stage 3) |
| 5 | 24,43 | 24,43  (-0,31) | Male | C-section | BPD, Cholestasis, IVH (grade 1), Sepsis, PDA, ROP (stage 3) |
| 6 | 24,57 | 590  (-1,39) | Female | Vaginal | IVH (grade 1), ROP (stage 1) |
| 7 | 24,86 | 800  (0,08) | Male | Vaginal | BPD, IVH (grade 4), PDA, Sepsis ROP (stage 3), |
| 8 | 26,00 | 415  (-5,24) | Female | C-section | IVH (grade 2), PDA,  ROP (stage 3) |
| 9 | 26,43 | 920  (-0,68) | Male | Vaginal | BPD, IVH (grade 2), PDA, Sepsis, ROP (stage 2) |
| 10 | 27,00 | 1235  (0,83) | Male | C-section | None |
| 11 | 27,29 | 1020  (-0,56) | Female | C-section | PDA, Sepsis |
| 12 | 27,29 | 965  (-0,93) | Female | C-cection | BPD |
| 13 | 27,43 | 930  (-1,31) | Female | C-section | PDA, ROP (stage 2) |
| 14 | 27,57 | 1230  (0,23) | Male | C-section | None |

*Abbreviations of perinatal data: GA – gestational age at birth. BW – birth weight. SDS – Standard deviations.
** Abbreviations of morbidities: BPD – Bronchopulmonary Dysplasia, IVH – Intraventricular hemorrhage (grade 1-4), NEC – Necrotizing Enterocolitis, PDA – Patent Ductus Arteriosus, Sepsis defined as: clinical symptoms + positive cultivation (unless coagulase-negative staphylococci, coryneform rods or mixed flora in which case CRP>20 or IL-6>1000 was required, ROP – Retinopathy of prematurity (stage 1-3)

**Supplementary Table S2 (online). Serum proteome profiling of fourteen extremely preterm babies**. (See separate excel-file)

**Supplementary Table S3 (online).** **Spearman correlation analysis of gestational age and retinopathy of prematurity with the assessed 448 serum proteins.** (See separate excel-file)

**Supplementary Table S4 (online).** **Spearman correlation analysis between the 448 serum proteins.** (See separate excel-file)
